# Two-dimensional and three-dimensional ultrasonographic diagnosis of congenital auricular anomalies

**DOI:** 10.3389/fmed.2025.1500895

**Published:** 2025-09-11

**Authors:** Huanyu Wang, Ke Lv, Xining Wu, Yixiu Zhang, Yunshu Ouyang, Yan Lv, Yulin Jiang, Yue Fan, Xiaowei Chen, Hua Meng

**Affiliations:** ^1^Department of Ultrasound, Peking Union Medical College Hospital, Peking Union Medical College, Chinese Academy of Medical Sciences, Beijing, China; ^2^Department of Obstetrics and Gynecology, Peking Union Medical College Hospital, Peking Union Medical College, Chinese Academy of Medical Sciences, Beijing, China; ^3^Department of Otorhinolaryngology, Peking Union Medical College Hospital, Peking Union Medical College, Chinese Academy of Medical Sciences, Beijing, China

**Keywords:** prenatal ultrasound examination, prenatal, fetus, congenital ear deformities, prenatal diagnosis

## Abstract

**Objective:**

To evaluate the role of prenatal ultrasound and three-dimensional ultrasound in the diagnosis of fetal congenital auricular malformations.

**Methods:**

The ultrasonographic features of 14 fetuses diagnosed with auricular malformations at Peking Union Medical College Hospital between May 2019 and May 2024 were retrospectively reviewed, and pregnancy outcomes were followed up.

**Results:**

The median gestational age at which congenital auricle deformities were detected by ultrasound was 24 weeks 5 days. Of the 14 cases, 5 had bilateral deformities, 5 had right-sided deformities, and 4 had left-sided deformities. The auricular abnormalities detected included microtia, low set ears, accessory auricles, abnormal ear helix and question mark ear, all of which were confirmed by postnatal follow-up or pathological examination after termination. Ten fetuses underwent three-dimensional ultrasound, which provided additional diagnostic details. Eight fetuses presented with other systemic deformities: three had facial deformities such as micrognathia, nasal bone absence, and facial cleft, while five had additional structure anomalies including diaphragmatic hernia, cardiac anomalies, and polydactyly. Genetic testing was performed in nine cases: one fetus had complete trisomy 18, one had Xp22.31 duplication, and seven had normal results. Six pregnancies were electively terminated, while eight fetuses were carried to term. Among the newborns, four had no significant hearing impairment, four others (three with microtia and one with a question mark ear) had hearing loss and required further reconstructive treatment.

**Conclusion:**

Congenital auricular deformities exhibit characteristic ultrasound features, and in most cases, prenatal diagnosis and evaluation can be achieved through ultrasound. This provides valuable information to support clinical decision-making and prenatal counseling.

## 1 Introduction

Congenital ear deformities can be categorized into morphological deformities and structural deformities based on the presence of cartilage developmental abnormalities. Structural ear deformities are often accompanied by malformations of the external auditory canal and middle ear, which not only affect the aesthetic appearance but may also lead to hearing impairments (1). Although fetal ear examination is not currently included in prenatal ultrasound screening guidelines, advancements in high-resolution prenatal ultrasound technology have made it possible to detect various congenital ear deformities, which is of considerable importance for prenatal counseling. At present, the literature on prenatal ultrasound screening for ear deformities consists mainly of individual case reports and retrospective reviews (2–5). Because of limitations in medical resources and a general lack of awareness among ultrasound practitioners in different hospitals, both misdiagnosis and missed diagnosis are relatively common.

In this study, we conducted a retrospective analysis of 14 fetuses with congenital auricular anomalies. The ultrasound features, associated anomalies, genetic findings, and clinical outcomes were systematically reviewed. The primary objective was to determine the diagnostic value of two-dimensional and three-dimensional prenatal ultrasound in identifying congenital auricular anomalies, with the broader aim of improving our understanding of this condition.

## 2 Materials and methods

### 2.1 Ethical approval

This study was reviewed and approved by Ethics Review Committee of Peking Union Medical College Hospital, Chinese Academy of Medical Sciences with the approval number: I-24YJ0986. And a waiver of informed consent was granted since no identifiable patient information was involved.

### 2.2 Patients

In total, 14 cases of congenital auricular malformations, diagnosed through routine prenatal ultrasound examinations between May 2019 and May 2024 at Peking Union Medical College Hospital or referred to our hospital for prenatal ultrasound evaluation, were retrospectively analyzed. All cases involved singleton pregnancies conceived naturally, with maternal ages ranging from 26 to 41 years (median: 30.0 years). The gestational age of ultrasound examination ranged from 21 to 32 weeks, with a median of 24 + 5 weeks.

### 2.3 Imaging acquisition

Ultrasound examinations were performed using a Voluson E10 machine (GE Healthcare), equipped with either a transabdominal two-dimensional (C5-1’ 1.0 ~ 5.0 MHz) or three-dimensional (RAB2-5 L, 2.0 ~ 5.0 MHz) convex array probe.

Scans were conducted by senior ultrasound physicians qualified in prenatal diagnostics. Routine systemic prenatal screening (level II prenatal ultrasound) was first performed to valuate overall fetal growth and development, detect any abnormalities, and access the fetal appendages. If any suspected malformations are detected, comprehensive fetal ultrasound is then conducted. The fetal auricles were then typically examined on the parasagittal plane of the temporal bone. In cases with suspected auricular abnormalities, multiplanar scans that included the coronal plane and cervical posterior transverse oblique planes were additionally performed. The position, symmetry, size, and shape of both auricles were carefully evaluated, along with detailed observation of the helix, antihelix, tragus, and earlobes. In suspected cases of microtia, the auricle was measured in the longitudinal view from the tip of the helix to the end of the lobe. For three-dimensional ultrasound, the transducer was positioned as perpendicular to the ear as possible, ensuring a sufficient volume of amniotic fluid and avoiding obstructions such as the umbilical cord or limbs. The scan was then switched to 3D surface mode, with adjustments made to the sampling box size and sector scan angle. The acquired 3D volume data were manipulated by rotating the X, Y, and Z axes, and adjusting the image grayscale, smoothness, and slice thickness to obtain a clear ear image.

### 2.4 Follow-up

After prenatal detection of congenital ear deformities, qualified cases were advised to undergo follow-up ultrasound every 3–4 weeks. After birth, immediate clinical follow-up by pediatricians or otolaryngologists was advised. For terminated pregnancies, pathological examination was conducted on the aborted fetuses. Genetic testing is recommended for the fetus based on patients’ medical history and gestational age, including karyotyping, whole-exome sequencing (WES), chromosomal microarray analysis (CMA), and copy number variation sequencing (CNV-seq).

## 3 Results

### 3.1 Ultrasound characteristics of congenital ear deformities

Prenatal ultrasound identified 14 cases of congenital ear malformations, with five cases being bilateral (35.71%) and nine cases unilateral (64.28%). Among the unilateral cases, five were on the right side, and four on the left. There were nine male fetuses (64.28%), and five female fetuses (35.71%). The main abnormalities detected on prenatal ultrasound included microtia, low-set ears, preauricular tags, Darwin notch on the helix, and question mark ears. Ten patients underwent three-dimensional ultrasound, which provided additional diagnostic detail (Table 1).

**Table 1:** Ultrasonographic features and prognosis of 14 cases of fetal auricle deformities.

ID	Age (years)	Gender of the fetus	Week of Gestation	Position	Features of ear deformities	Associated deformities	Outcome
1	35–40^*^	Male	33w + 6d	Bilateral	Grade I microtia	Diaphragmatic hernia, possible left pelvic kidney, polydactyly on the right hand, polyhydramnios	Hearing impairment after birth
2	>40	Female	24w + 5d	Bilateral	Grade II microtia	Micrognathia, single umbilical artery, lobed placenta, polyhydramnios	Induced labor
3	25–30^*^	Male	30w + 1d	Bilateral	Grade III microtia	Micrognathia, nasal bone absence, polyhydramnios	Induced labor
4	30–35^*^	Male	23w + 2d	Bilateral	Grade II microtia	–	Induced labor
5	25–30^*^	Female	23w + 5d	Right	Grade III microtia	Ventricular septal defect	Induced labor
6	30–35^*^	Female	22w + 2d	Right	Grade II microtia	Ventricular septal defect	Hearing impairment after birth
7	30–35^*^	Male	26w + 2d	Right	Grade II microtia	–	Induced labor
8	25–30^*^	Male	24w + 6d	Right	Grade I microtia	–	Induced labor
9	35–40^*^	Male	31w	Left	Grade I microtia	Intrauterine growth restriction, placental calcifications, battledore placenta, high coiled umbilical cord	Hearing impairment after birth
10	25–30^*^	Female	21w + 2	Bilateral	Accessory auricles	–	Normal hearing present at birth
11	25–30^*^	Male	22w + 6	Left	Accessory auricles	–	Normal hearing present at birth
12	25–30^*^	Female	32w	Left	Accessory auricles	Diaphragmatic hernia, polyhydramnios	Normal hearing present at birth
13	25–30^*^	Male	22w	Left	Darwin notch on helix	-	Normal hearing present at birth
14	25–30^*^	Female	28w + 3	Right	Question mark ear	Facial cleft	Hearing impairment after birth

^*^25–30:>25 and ≤ 30. 30–35:>30 and ≤ 35. 35–40:>35 and ≤ 40.

#### 3.1.1 Microtia

**Figure 1:**
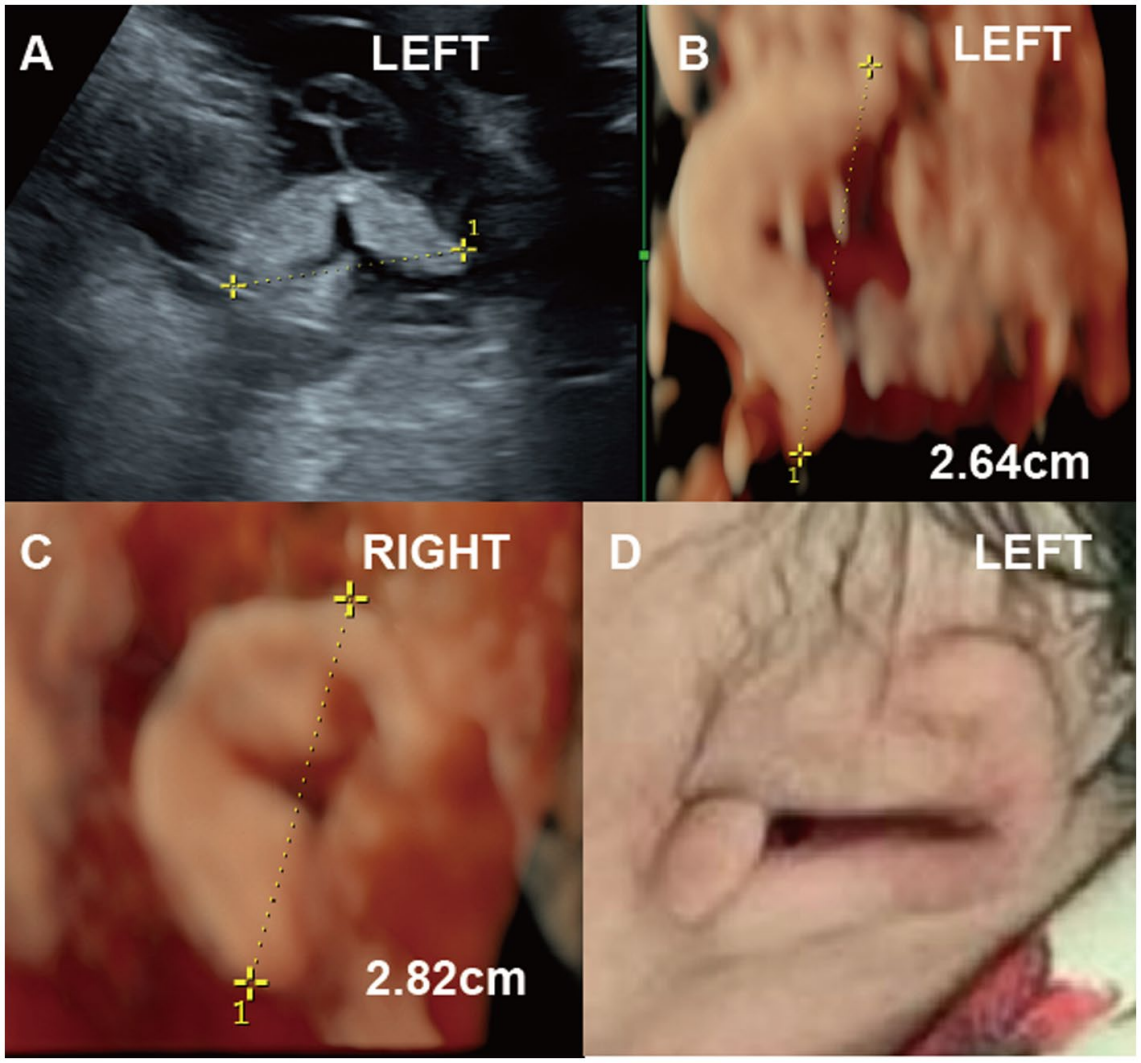
(Case 9). A pregnant woman at 22 weeks’ gestation underwent ultrasound, which revealed fetal growth restriction and abnormalities in the left ear **(A-C)**. By 30 weeks, two-dimensional ultrasound measured the left ear length at 2.6 cm, while three-dimensional ultrasound showed unclear ear structures **(D)**. Postnatal photographs confirmed Grade I microtia of the left ear, characterized by an underdeveloped helix, absent lobule, antihelix, and fossa, along with a poorly defined pinna. A newborn hearing test confirmed hearing impairment.

**Figure 2:**
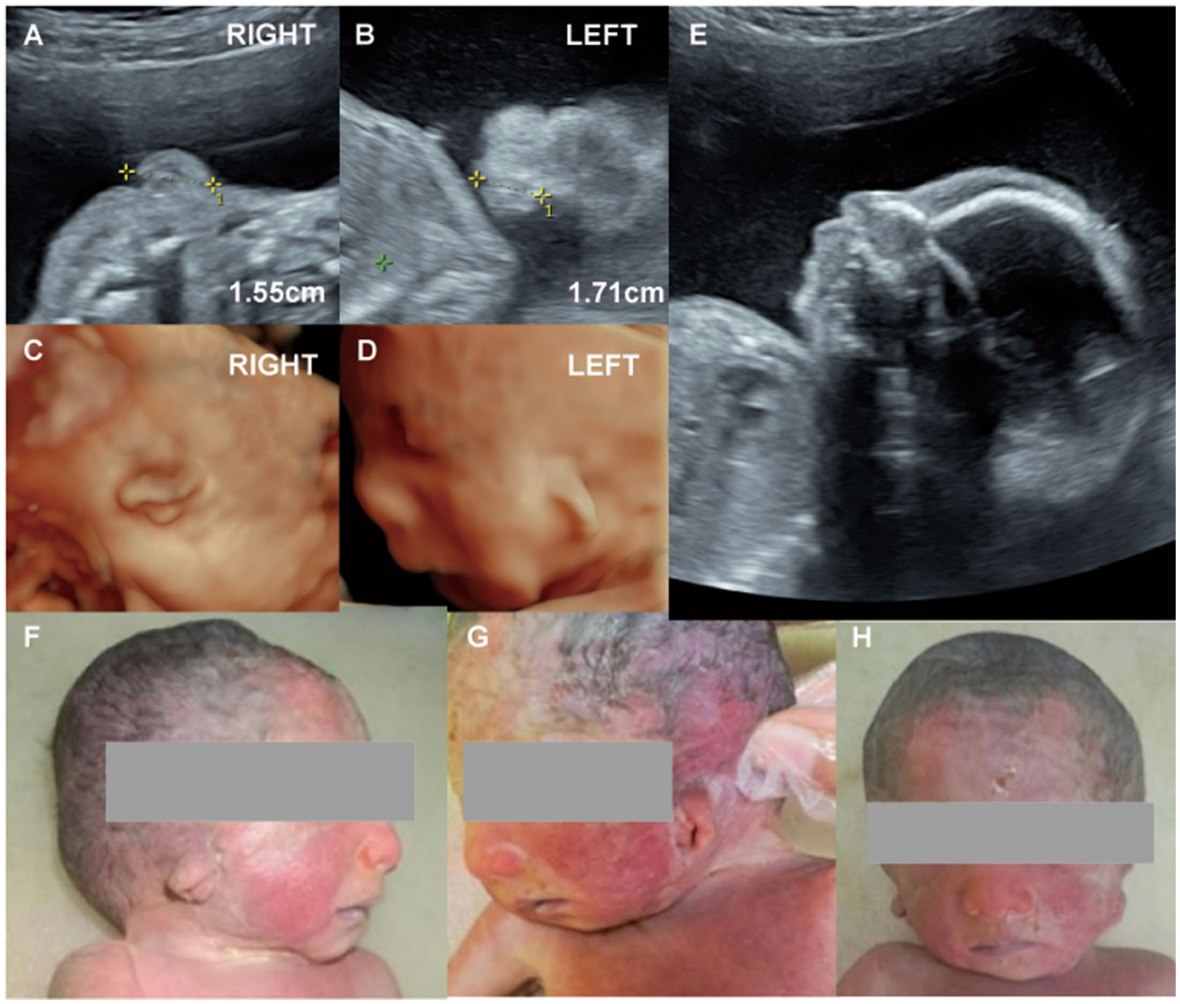
(Case 2). **(A,B)** At 23 weeks’ gestation, routine ultrasound revealed bilateral microtia. **(C,D)** Three-dimensional imaging showed that both ears were small, misshapen, and low-set, with rotation of the right ear. **(E)** Additional findings included micrognathia, a single umbilical artery, and polyhydramnios. Genetic testing was normal. **(F-H)** Labor was induced at 26 weeks, and postnatal examination confirmed bilateral Grade II microtia.

**Figure 3:**
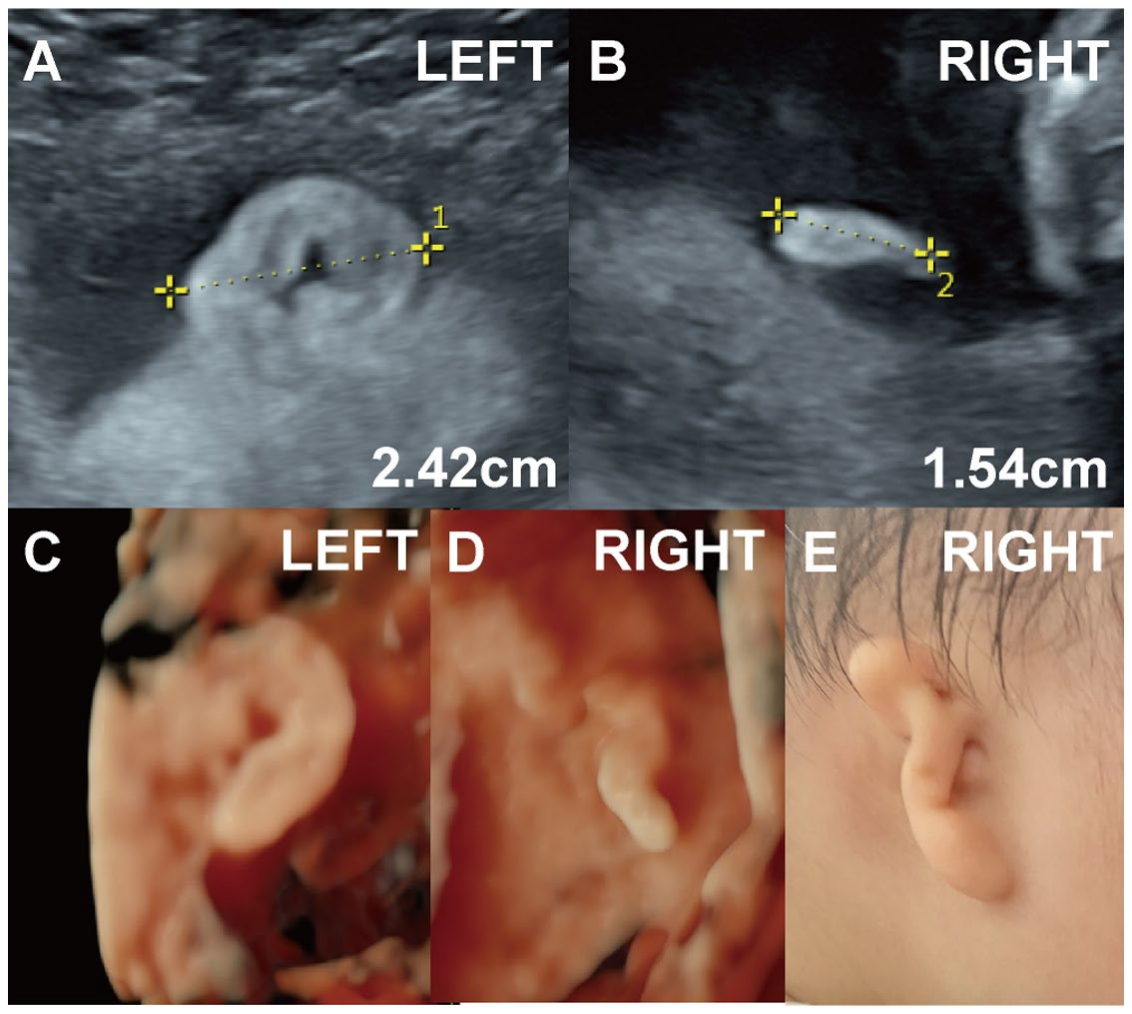
(Case 6). **(A,B)** A systemic ultrasound at 22 weeks revealed an abnormal right ear. Two-dimensional imaging showed a right ear length of approximately 1.5 cm with a slightly elevated, ribbon-like, peanut-shaped helix. Genetic testing was normal. **(C,D)** Three-dimensional ultrasound was also performed. **(E)** The pregnancy resulted in the full-term delivery of a baby girl with right-sided Grade II microtia and external auditory canal atresia. Hearing tests at 11 months confirmed conductive hearing loss in the right ear.

There were nine cases of microtia (Cases 1–9), including four cases of bilateral microtia, four cases of right-sided microtia, and one case of left-sided microtia. Among these, three were classified as grade I, four as grade II, and two as grade III microtia. Two cases were also accompanied by low-set ears (Figures 1–3).

#### 3.1.2 Accessory auricle

**Figure 4:**
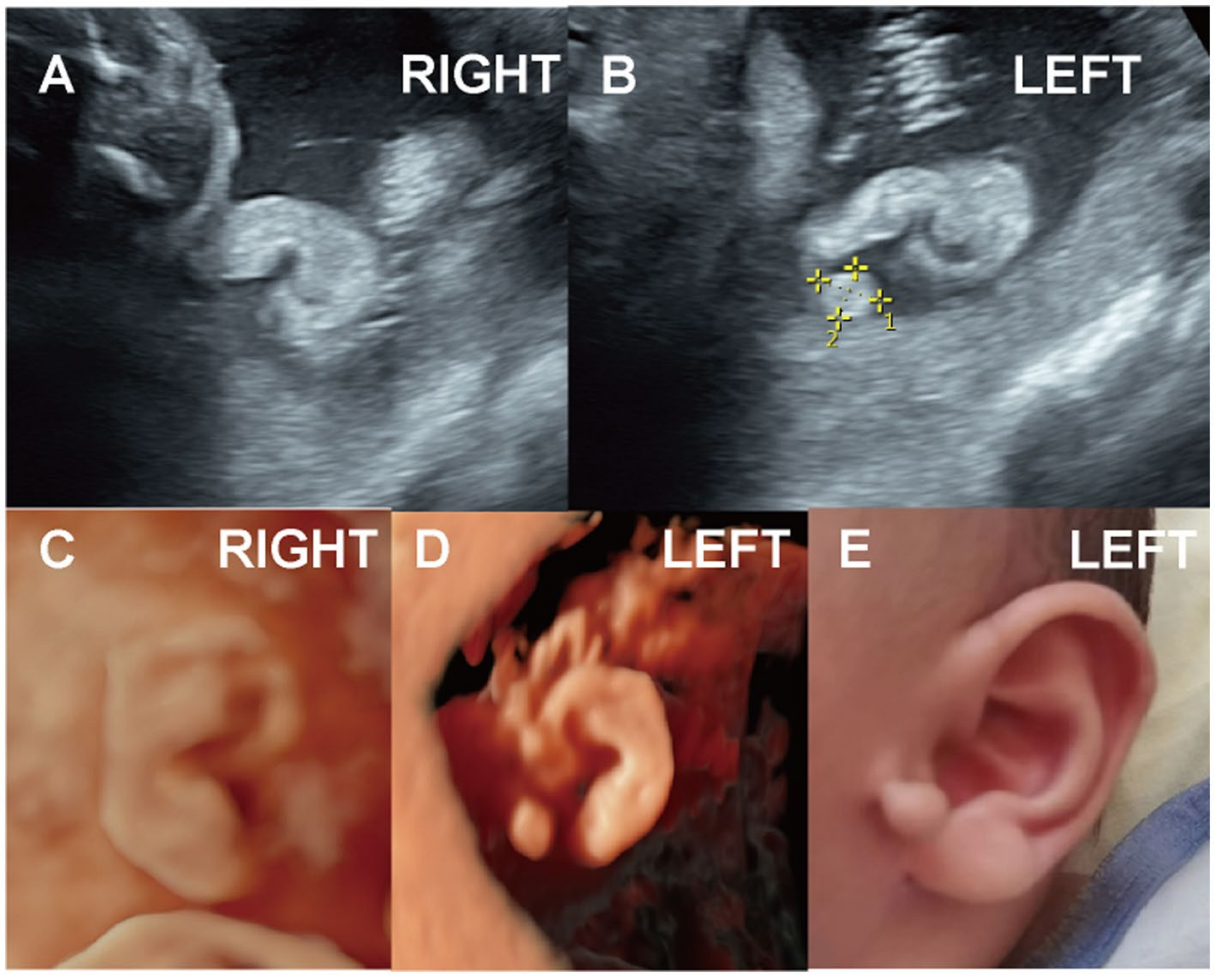
(Case 11). A pregnant woman, whose husband had an accessory auricle on his right ear, underwent ultrasound at 22 weeks 6 days. **(A-D)** The scan revealed a moderately echogenic accessory auricle on the left ear of the fetus, measuring 0.8 × 0.6 cm. No other abnormalities were noted, and genetic testing was not performed. **(E)** A full-term baby boy was delivered with an accessory auricle located below the left earlobe. Hearing test results were normal.

Three cases involved accessory auricles, including one case of bilateral malformation (Case 10), and two cases of left-sided accessory auricles (Figure 4, Case 11; Case 12). These ranged in size from approximately 0.5 to 0.8 cm, and were located in front of or below the tragus.

#### 3.1.3 Others

**Figure 5:**
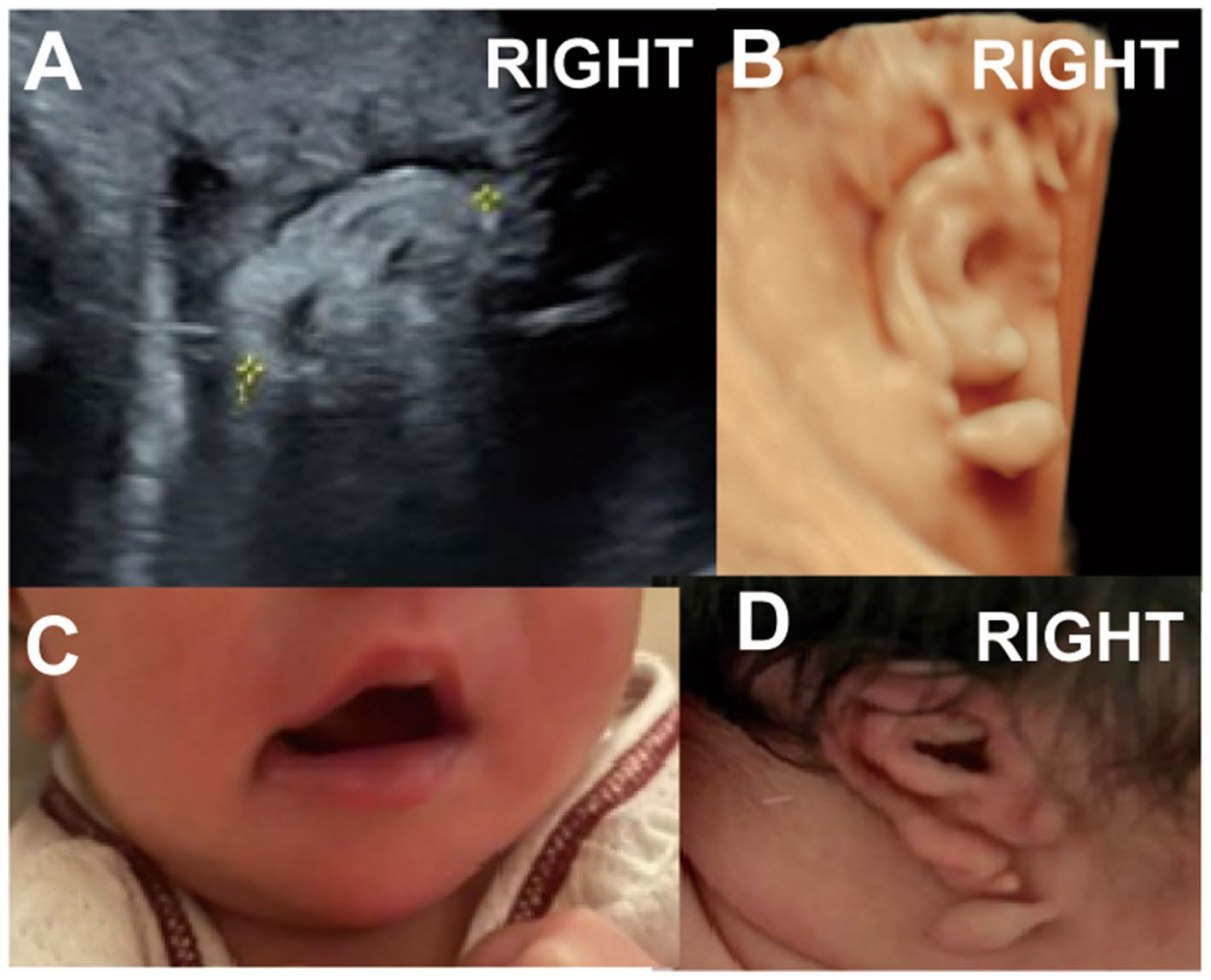
(Case 14). At 24 weeks’ gestation, ultrasound indicated a right ear deformity, prompting a referral. **(A,B)** By 28 weeks, the left ear measured 2.6 cm with normal morphology, while the right ear measured 2.1 cm and showed abnormal structure with a lump beneath the earlobe. **(C,D)** A full-term baby girl was delivered with a question mark ear deformity and a lateral facial cleft on the right side. Hearing tests showed elevated ABR response thresholds, delayed wave latencies, and a possibly prolonged T-V interval on the right side.

One case presented with a Darwin notch on the helix, and one case exhibited a right-sided question mark ear (Figure 5, Case 14), both identified during prenatal ultrasound examination.

### 3.2 Combined facial deformities and other structural abnormalities

Among the 14 cases of ear deformities, three were associated with facial abnormalities. Two cases of bilateral microtia were accompanied by micrognathia (Cases 2 and 3), with one of these also showing nasal bone deficiency (Case 3). One case of right-sided question mark ear was associated with an ipsilateral facial cleft (Figure 5, Case 14).

Five cases (35.71%) were complicated by additional structural anomalies, including two cases with diaphragmatic hernia (Case 1, Case 12), two cases with cardiac anomalies (Case 5, Case 6); one with polydactyly (Case 1); and one with intrauterine growth restriction (Case 9). Additionally, polyhydramnios were observed in four cases.

### 3.3 Results of genetic examinations

Genetic testing was performed in nine of the 14 cases with congenital auricular anomalies. One case of bilateral microtia was diagnosed with complete trisomy 18 (Case 5); and one case with unilateral microtia was found to have a 1.6 Mb duplication on Xp22.31. The remaining seven cases showed normal karyotype results.

### 3.4 Follow-up

Of the 14 cases with ear deformities, six resulted in pregnancy termination, with postnatal pathological examination confirming the prenatal ultrasound diagnoses. Among the eight cases carried to term, four exhibited hearing impairments comprising three cases of microtia and one case of question mark ear, while the other four had normal hearing. The latter group included three cases of accessory auricles and one case of helical rim cleft.

### 3.5 Family history

Among the cohort of 14 pregnant women, two (Cases 4 and 10) had a documented history of adverse pregnancy outcomes involving fetuses diagnosed with microtia. In Case 11, the father of the fetus was diagnosed with accessory auricles. Notably, one of these women (Case 10) underwent familial genetic testing, and no abnormalities were detected through WES of the family.

## 4 Discussion

During the embryonic period, the development of the ear originates from the first branchial groove, the first branchial arch, and the second branchial arch. By the sixth week of embryonic development, the basic outline of the ear begins to form. Between the 13th and 14th weeks, the six auricular hillocks fuse to create the rudimentary ear, which gradually ascends above the level of the inner canthi as the mandible grows (6). By the 20th week of gestation, the anatomical features of the ear closely resemble those of an adult, and the length of both ears shows a linear correlation with gestational age (2). Between 20 and 24 weeks of gestation, the relatively larger volume of amniotic fluid results in optimal visualization of the ear structure. On ultrasound, the ear appears moderately echogenic, with a highly echogenic edge forming a characteristic “C” shape and an uneven surface. The center is concave, forming the external auditory canal, while the helix can be seen along the outer edge, gradually merging into the smoother earlobe. By the third trimester, structures such as the antihelix, scapha, cymba conchae, triangular fossa, tragus, and antitragus become increasingly distinguishable. Three-dimensional ultrasound enables multi-angle visualization of the auricle, allowing for more detailed assessment of its morphology, position, and size (7, 8). In this study, the median gestational age at which ear deformities were detected was 24 weeks and 5 days, aligning with findings from previous research (9).

The incidence of congenital ear deformities is approximately 1 in 6,000 to 1 in 6,830 births (8). During the embryonic period, pathogenic agents, drugs, or genetic factors can disrupt normal development and lead to ear malformations (10). With the advancement of high-resolution prenatal ultrasound technology, various ear deformities—such as microtia, anotia, and accessory auricles—can now be detected (3, 8). Congenital microtia is defined as an ear length that is more than two standard deviations below the mean for the corresponding gestational age (4, 11), typically accompanied by abnormal or absent ear morphology. Microtia is commonly classified into three grades: Grade I, in which most anatomical structures of the auricle are present but smaller in contour compared with the normal side; Grade II, in which the auricle is reduced to approximately half to two-thirds of normal size, with major features such as the helix absent; and Grade III, the most common type, in which most anatomical structures are unrecognizable or missing, leaving behind residual structures that appear sausage-shaped, peanut-shaped, or boat-shaped, often accompanied by atresia or absence of the external auditory canal (12). Therefore, prenatal evaluation of the fetal ear should involve not only measuring its length but also observing its morphology. In this study, of the nine microtia cases, unilateral involvement was more frequent than bilateral, with a higher prevalence on the right side—findings that are consistent with previous research (13, 14). Microtia is often associated with atresia or stenosis of the external auditory canal, and literature reports indicate that more than 90% of affected individuals suffer from conductive hearing loss. While visualizing the external auditory canal via ultrasound is generally considered difficult, some researchers have successfully identified the lateral segment using two- and three-dimensional imaging techniques (15). Beyond microtia, prenatal ultrasound can also detect other congenital ear deformities such as abnormal positioning and accessory auricles. Low-set ears are defined as ears whose upper borders lie below the line connecting the outer canthi of both eyes to the occiput (10). An accessory auricle is a vestigial remnant resulting from incomplete development of the anterior ear during embryogenesis; it can appear anywhere within the curved triangular area extending from in front of the tragus to the line between the mouth corner and ear, with the region immediately anterior to the tragus being most common (16). Question mark ear (also known as ear condyle syndrome or auriculo-condylar syndrome) is a distinct malformation marked by a cleft between the helix and earlobe in the mid-lower ear, which divides the auricle into upper and lower segments with helix discontinuity. It is attributed to developmental anomalies of the first and second pharyngeal arches and is often associated with a broad spectrum of phenotypes, including ear deformities, mandibular hypoplasia, and other craniofacial abnormalities (17).

Congenital ear deformities are often associated with craniofacial and other systemic anomalies. In this study, children with question mark ear deformities also presented with facial clefts and hearing loss on the affected side. A facial cleft results from disrupted fusion between the maxillary and mandibular processes at the corner of the mouth, leading to incomplete closure and outward displacement of the oral commissure. This condition frequently co-occurs with ear deformities, micrognathia, and other anomalies. Therefore, when ear deformities are detected during prenatal screening, careful evaluation of the jaw and corners of the mouth is warranted. Conversely, when facial clefts or micrognathia are observed, the fetal ears should be closely examined to avoid misdiagnosis. In this study, two fetuses also exhibited micrognathia. Takano et al. (18) investigated the relationship between the severity of congenital microtia and mandibular malformations, finding that patients with a coronoid process ratio (compared with the healthy side) of less than 0.85 frequently had Grade III microtia. Ear deformities are also commonly associated with cardiovascular and skeletal anomalies, which may reflect underlying syndromic conditions. In our cohort, five fetuses exhibited structural abnormalities in other systems. The likelihood of associated malformations appears to correlate with the severity of the ear deformity; more severe auricular underdevelopment is often linked to higher rates of comorbid anomalies. This may be due to teratogenic influences affecting multiple developmental pathways. As such, when anomalous ears are identified, careful examination for additional congenital anomalies—particularly those that may be subtle or easily missed, such as renal dysgenesis—is strongly recommended.

Congenital ear deformities are also frequently associated with genetic abnormalities. Previous studies have shown that microtia may be linked not only to triploid but also to non-triploid chromosomal abnormalities such as trisomy 13, trisomy 18, and trisomy 22. Growth parameters such as ear length, width, and surface area serve as useful indicators for screening chromosomal abnormalities (4, 19, 20). Beyond chromosomal abnormalities, mutations in certain genes are also closely associated with external ear malformations (21). For instance, *HOXA2* is a critical transcription factor involved in ear development (22). Mutations in *HOXA2* can cause microtia and preauricular tags (23). A characteristic auricular phenotype is also observed in clinical syndromes caused by specific genes. For example, CHARGE syndrome driven by *CHD7*, and Kabuki syndrome driven by *MLL2/KMT2D/KDM6A* both demonstrate fetal ear developmental abnormalities (24, 25). In our study, one fetus was found to have complete trisomy 18 syndrome. Low-set ears have also been associated with fetal chromosomal abnormalities. Therefore, prenatal ultrasound detection of ear developmental anomalies can serve as an important prompt for genetic testing and clinical decision-making (26). In this cohort, the overall prognosis for the nine children with microtia was poor; hearing impairment was observed in all three newborns who were delivered. However, it is important to note that a negative genetic testing result does not necessarily exclude an underlying genetic defect. Limitations may exist in the testing methodology or expertise, and results can also depend on the sample obtained. In Case 2, both karyotyping and WES revealed no abnormalities; nevertheless, this cannot entirely rule out underlying genetic defects. Case 4 was diagnosed with bilateral microtia and absent external auditory canals in the current pregnancy, following a history of microtia in a previous pregnancy. WES analysis of tissue in both induced deliveries detected no abnormalities.

One limitation of this study is that only one fetus underwent prenatal magnetic resonance imaging (MRI). This imaging technique holds significant diagnostic value in assessing fetal external ear deformities and serves as an effective complement to ultrasound, particularly for detecting external auditory canal atresia. The absence of a high T2-weighted imaging signal in the external auditory canal on MRI may indicate atresia. Thus, when ultrasound identifies ear deformities, further MRI evaluation is recommended to confirm the presence of associated external auditory canal abnormalities.

In conclusion, level II or advanced prenatal ultrasound, including 3D modalities, are valuable for identifying fetal ear deformities and associated craniofacial anomalies. For experienced sonographers, a thorough understanding of the sonographic features of these abnormalities is crucial for accurate prenatal diagnosis, which provide essential information for clinical management and prenatal counseling.

## Data Availability

The original contributions presented in the study are included in the article/supplementary material, further inquiries can be directed to the corresponding authors.
